# Robotic keyhole access to the semicircular canals for vestibular implantation: an anatomical feasibility study

**DOI:** 10.1007/s00405-025-09695-0

**Published:** 2025-11-04

**Authors:** Philipp Aebischer, Tom Gawliczek, Elke Loos, Franca Wagner, Stefan Weder, Marco Caversaccio, Nils Guinand, Raymond van de Berg, Georgios Mantokoudis

**Affiliations:** 1 Hearing Research Laboratory, ARTORG Center for Biomedical Engineering Research, University of Bern, Murtenstrasse 50, 3008 Bern, Switzerland; 2 Department of Otorhinolaryngology, Head and Neck Surgery, Inselspital University Hospital, Bern, Switzerland; 3 Department of Diagnostic and Interventional Neuroradiology, Cantonal Hospital Aarau, Aarau, Switzerland; 4 Department of Otorhinolaryngology–Head and Neck Surgery, School for Mental Health and Neuroscience, Faculty of Health Medicine and Life Sciences, Maastricht University Medical Center, Maastricht, Netherlands; 5 Department of Otorhinolaryngology, Head and Neck Surgery, University Hospitals Leuven, Leuven, Belgium; 6 Department of Neurosciences, Research Group Experimental Oto-Rhino-Laryngology (ExpORL), KU Leuven, Leuven, Belgium; 7 Division of Otorhinolaryngology–Head and Neck Surgery, Department of Clinical Neurosciences, Geneva University Hospitals, University of Geneva, Geneva, Switzerland

**Keywords:** Vestibular implant, Robotic keyhole access, Semicircular canal, Image-guided surgery, Temporal bone anatomy, Electrode insertion, Minimally invasive surgery

## Abstract

**Background:**

Vestibular implants are a promising treatment for patients with severe bilateral vestibulopathy. However, precise and minimally invasive access to the semicircular canals remains a key surgical challenge.

**Objective:**

To evaluate the anatomical feasibility of robotic keyhole access to the three semicircular canals for vestibular implantation using image-guided drilling.

**Methods:**

High-resolution computed tomography scans from 30 ears were analyzed to simulate drill trajectories to the superior, lateral, and posterior semicircular canals. Trajectories were evaluated for surgical accessibility, safety margins relative to critical structures, and approach angles suitable for electrode insertion. Mutually optimized entry points within a simple cortical mastoidectomy were identified.

**Results:**

Safe access to the three canals was feasible in all cases. The lateral and posterior canals were consistently accessible at favorable semicircular canal entry angles. The superior canal required steeper semicircular canal entry angles but remained within acceptable limits. In all cases, three suitable trajectories could be planned to originate from a single 15 mm retroauricular mastoidectomy region.

**Conclusion:**

Robotic keyhole access to the semicircular canals is anatomically feasible across a range of patient anatomies. These findings support the clinical potential of minimally invasive, image-guided vestibular implantation.

## Introduction

Surgical access to the semicircular canals (SCC) of the labyrinth is primarily performed in cases of semicircular canal dehiscence, intractable benign paroxysmal positional vertigo (BPPV), or in patients undergoing vestibular implantation.

However, these procedures are notably challenging due to several key factors: they are rare, resulting in limited surgeon familiarity; there is a risk of hearing loss and loss of vestibular function caused by perilymph or endolymph leakage, inflammation, or surgical trauma [4, 5]; the procedure often requires a large mastoidectomy via a transmastoid approach, resulting in a larger retroauricular incision and scar; and the precise placement of fenestration with respect to the ampulla is challenging because the canals are deeply embedded in the dense labyrinthine bone [21]. As a result, precise placement of vestibular electrodes at their desired location inside the ampulla remains difficult. Due to the lack of direct visual access, intraoperative imaging is frequently employed to guide and verify electrode positioning [14, 19].

Despite these technical challenges, vestibular implants offer a promising solution for restoring vestibular function in patients with severe vestibulopathy [8, 9]. Two main types of implants are under investigation: semicircular canal-stimulating implants, which use external processors and sensors (developed by research groups in Geneva-Maastricht, Baltimore and Seattle) to restore the semiciruclar canal’s function based on the movements of the patients’ head [6, 7, 18], and implants designed for otolithic stimulation, to enhance postural reflexes [16]. These technologies represent significant advancements in treating vestibular disorders, although precise surgical access and electrode positioning remain critical challenges [15].

In recent years, image-guided robotic keyhole access has been introduced into clinical practice for cochlear implantation [3, 22]. In addition to its minimally invasive nature, the procedure enables submillimeter precision in reaching targets within the temporal bone [17]. This level of accuracy makes it particularly well suited for vestibular access, where the absence of clear anatomical landmarks poses a challenge for precisely creating the canal fenestration. More recently, robot-assisted systems have also been developed for electrode insertion, enabling slow and highly accurate placement with positional precision down to tenths of a millimetre [1].

Although these technologies have become established in cochlear implant surgery, their potential for other inner ear procedures remains largely unexplored. In this study, we comprehensively evaluated the feasibility of robotic keyhole access for intralabyrinthine vestibular implantation. Using high-resolution computed tomography data from 30 temporal bones, we assessed the anatomical accessibility of all three canals and evaluated the spatial proximity to surrounding critical structures. Particular focus was placed on achieving favorable SCC entry angles and identifying mutually compatible drill entry points, to support safe and feasible clinical implementation of the procedure.

**Fig. 1:**
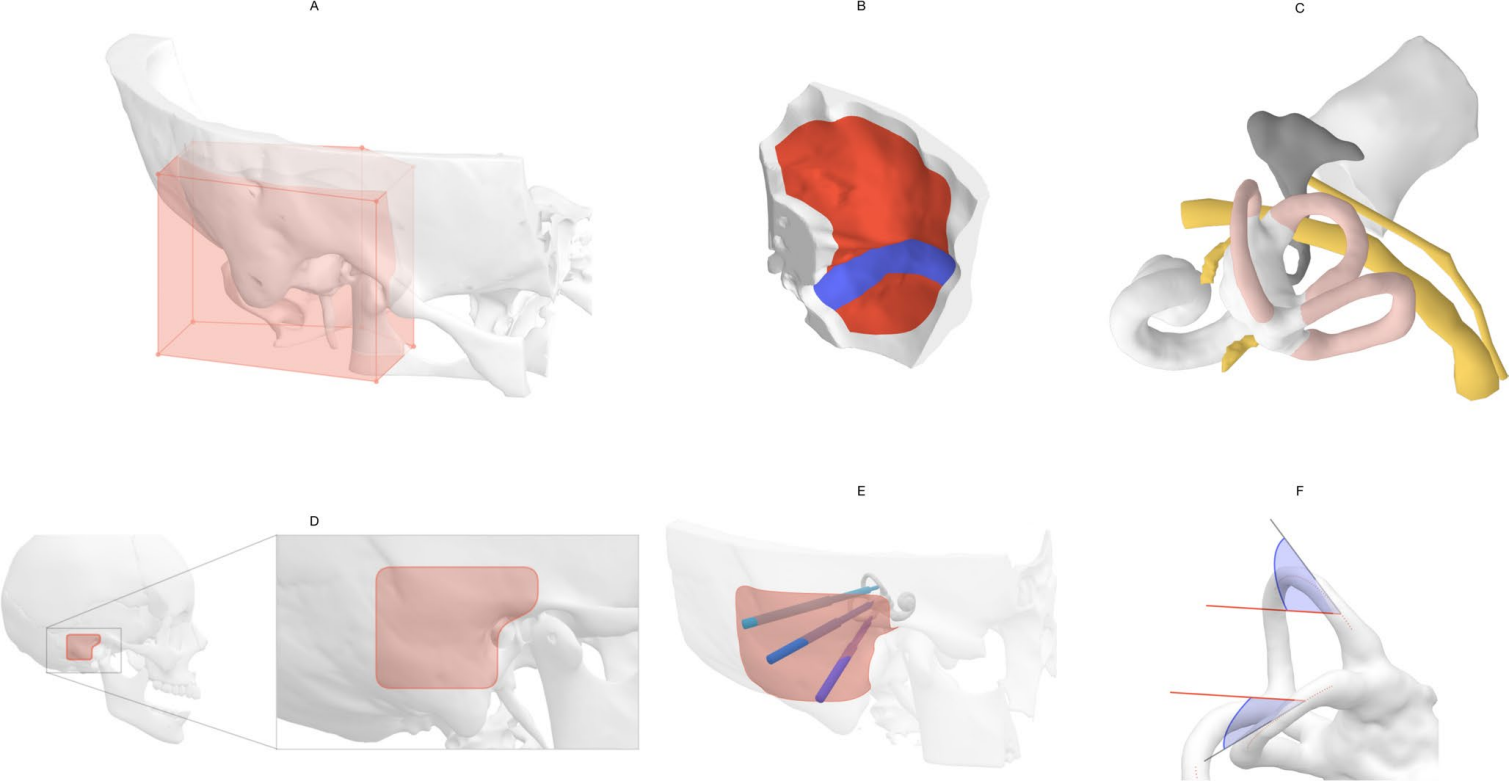
Data preparation and preprocessing. (A) The temporal bone segmentation was cropped around the region of interest. (B) The lateral skull base and the bounding surface of the sigmoid sinus were generated from the temporal bone mesh and sinus centreline imported from Otoplan. (C) The semicircular canals, facial nerve, chorda tympani, and external auditory canal were directly imported from Otoplan. Additionally, manual segmentation of the superior vestibular nerve and posterior ampulary nerve were imported. (D) A $30\,\text {mm} \times 30\,\text {mm}$ retroauricular area, vertically centred posterior to the external auditory canal, defined the drill entry point area. Note the anterosuperior extension which helps with orientation. (E) Visualisation of three drill canals through the entry point area. (F) The approach angles (here visualised with the planned cochlear implant drill direction) were measured with respect to the tangent of the canal centreline at the target site

## Methods

The goal of image-guided robotic vestibular implantation is to create three drill trajectories to the semicircular canals (SCC). These trajectories should enter the canals at favorable, near-tangential angles. This enables electrode insertion along the drill path and the canal toward the targeted ampullary region. To ensure safety, trajectories must maintain adequate clearance from critical anatomical structures such as the facial nerve, lateral skull base, and sigmoid sinus.

For clinical applicability, the drill entry points on the mastoid surface must be reasonably close to one another. A simple cortical mastoidectomy is required to accommodate the excess implant cables. Ideally, all three trajectories should be mutually optimized to originate within the same mastoidectomy and enter the canals at angles that allow smooth advancement of the electrode up to the target site.

This study investigated the anatomical feasibility of such keyhole access and evaluated whether appropriate entry points and approach angles could be identified across a large cohort of individuals with standard anatomical inner ear features.

### Demographics

Pre-operative photon-counting CT (PCCT) data from 30 ears scheduled for inner-ear surgery were analysed. The cohort included 13 female and 17 male ears, aged $56 \pm 19$ years (range 16–87 years). Scans comprised 16 left and 14 right temporal bones.

### Image acquisition

Images were acquired on a NAEOTOM Alpha photon-counting CT system (Siemens Healthineers, Erlangen, Germany). The imaging protocol provided an in-plane resolution of $0.195\,\text {mm} \times 0.195\,\text {mm}$ and a slice thickness of $0.2\,\text {mm}$. This high spatial resolution allowed detailed visualization of the osseous labyrinth, adjacent nerves, and other critical anatomical structures relevant to trajectory planning.

### Image segmentation

All radiographic images were processed using Otoplan V5 (CASCINATION AG, Bern, Switzerland), which provides automated segmentation of the temporal bone, cochlea, and semicircular canals, minimizing the impact of inter-operator variability. The facial nerve and chorda tympani were segmented semi-automatically by manually marking centreline points, from which the software automatically generated cross-sectional contours along the course of the nerve. Segmentation extended across the entire anatomical course relevant to this study.

Robotic cochlear implantation planning was also performed using the software, which automatically proposed an optimal drill trajectory. In two cases where the automatically generated trajectory bypassed the facial recess, manual adjustments were made to ensure a clinically viable drill path. Planning was carried out by two experts experienced in robotic cochlear implantation.

Finally, the superior vestibular nerve and posterior ampulary nerve were manually segmented in 3D Slicer (https://www.slicer.org/ [11]), and a nerve stimulation target selected for each canal by an expert in the segmentation of the vestibular system.

### Robotic technology

For this study, we assumed dimensional parameters corresponding to the HEARO robotic system (CASCINATION AG, Bern, Switzerland), which is a clinically available platform with published technical specifications for image-guided keyhole drilling in temporal bone surgery. Specifically, the drill bit diameter and stepped profile were used to define safety margins and trajectory clearance in our simulations. Apart from these dimensions, the analysis was kept agnostic to the device hardware and control software, and the approach should be transferable to other robotic or navigation-assisted platforms with comparable submillimetric accuracy. The HEARO system integrates preoperative imaging, segmentation, and trajectory planning with image-guided robotic drilling, achieving a mean positional accuracy of $0.15\,\text {mm}$ in clinical cochlear implantation. These characteristics were used to define the safety margins in our simulations and to guide the feasibility evaluation of vestibular access within the studied anatomical constraints.

### Data processing

Planning files from Otoplan and 3D Slicer were imported into Python 3.12 for further processing using a custom script. All surface-based segmentations were converted to Trimesh format. To standardize mesh sizes across all cases, a vertical triangular prism around the region of interest was extracted from each temporal bone model. The data processing steps are visualised in Fig. 1. An outer surface of the temporal bone excluding mastoid air cells was generated by remeshing the convex hull to uniformly spaced vertices, and projecting the vertices to the nearest surface point of the original mesh. This process of uniform remeshing and projection was repeated until the mesh converged to a stable geometry. All meshes were remeshed in instant meshes (https://igl.ethz.ch/projects/instant-meshes/ [10]), with a targeted face size of $0.1\,\text {mm}^{2}$. For consistency in analysis and visualisation, geometries of left ears were mirrored.

#### Lateral skull base

The lateral skull base was computed procedurally from the temporal bone segmentation. The mesh face positioned superior of the the inner ear centre was identified, and all connected faces within a bounding box around the region of interest were selected to represent the lateral skull base surface. For the sigmoid sinus, we procedurally casted rays along the sinus centreline uniformly in all directions to find intersections with the temporal bone surface, and computed the alphashape of the resulting point cloud with an alpha of 0.1. The mesh of the sigmoid was subtracted from the lateral skull base. An example of the resulting surfaces are shown in panel (B) of Fig. 1.

#### Drill entry point area

We analyzed drill trajectories originating from a designated surface on the mastoid bone. Specifically, a $30\,\text {mm} \times 30\,\text {mm}$ retroauricular region was defined as the drill *entry point area*. This area is oriented in the sagittal plane and positioned on the outer surface of the temporal bone. A visual example of the region is shown in Fig. 1, panel (D, E). The region was centred vertically and aligned horizontally with the anterior edge to the centre of the ear canal. A superoanterior extension of $10\,\text {mm} \times 10\,\text {mm}$ was added towards the zygomatic process, which also serves for visual orientation purposes. Within the drill entry point area, 5000 evenly spaced points were generated on the surface of the temporal bone for subsequent analysis.

#### Drill targets

The centreline of each semicircular canal was computed by voxelising the mesh on a $0.1\,\text {mm}$ grid, skeletonising according to the method of Lee et al. [13], and smoothing into a spline curve. The *electrode target point* was defined as the point on the centreline closest to the manually annotated nerve stimulation target. The *fenestration site* was defined as the targeted end point of a drill trajectory, positioned on the canal centreline. The default fenestration site was defined as the point $2\,\text {mm}$ from to the electrode target point along the canal centreline.

#### Distance computation

Signed distance fields were computed for each anatomical structure on a rectangular grid with a spacing of $0.125\,\text {mm}$. For each candidate trajectory, points were sampled at the same resolution along the path from the drill entry point on the mastoid surface to the fenestration site within a semicircular canal. At each sampled point, the distance to surrounding structures was determined by linear interpolation of the corresponding quantized distance fields. The geometric clearance at each sampled point to each structure was calculated by subtracting the drill radius, using a value of $0.5\,\text {mm}$ for the tip, with a first step up to $0.6\,\text {mm}$ after $4\,\text {mm}$ and a second step up to $1.25\,\text {mm}$ after $14\,\text {mm}$ [20, 22]. Finally, the safety margin of a trajectory was defined as the minimum clearance along its path.

### Fenestration size

**Fig. 2:**
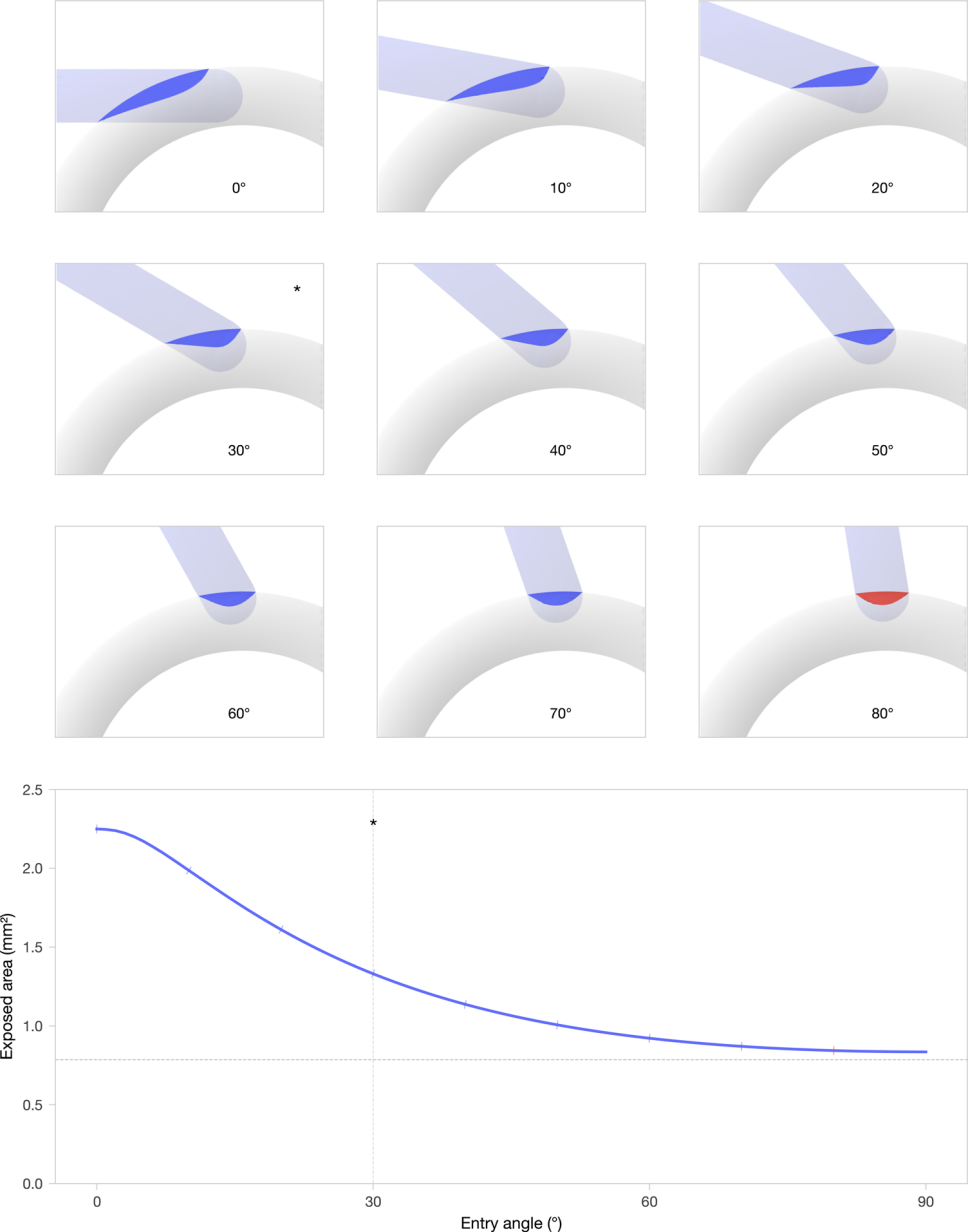
Top: visualisation of the exposed canal surface in an idealized geometry for different semicircular canal (SCC) entry angles ranging from $0^{\circ }$ to $80^{\circ }$. Angles above $80^{\circ }$ were considered inaccessible, as an inserted electrode may risk following a retrograde direction. Bottom: Exposed surface area as a function of SCC entry angle. The ideal SCC entry angle of $30^{\circ }$ is indicated with an asterisk (*)

Semicircular canals have an internal height ranging from $1.0\,\text {mm}$ to $1.2\,\text {mm}$ [12]. The robotic system used in this study features a $1\,\text {mm}$ spherical drill tip, similar in size to the canal lumen. Drilling into a semicircular canal exposes part of the canal surface. When the drill enters at a right angle, the resulting opening is approximately circular. As the SCC entry angle becomes shallower, however, the exposed area becomes increasingly elongated, as illustrated in Fig. 2 for an idealized geometry. A tangential approach is therefore undesirable, as it results in an excessive exposure of the canal lumen. The bottom panel in Fig. 2 shows the exposed surface area as a function of the SCC entry angle. Based on this relationship, we proposed an ideal SCC entry angle of $30^{\circ }$, representing a suitable trade-off between a limited exposure of the canal lumen and achieving a straight electrode trajectory.

### Mutual trajectory optimization

The drill three entry points must be reasonably close to one another, as the individual electrodes branch from a single implant lead. Current implant designs require a shared cortical mastoidectomy to receive the excess electrode cables. Minimizing the distance between the entry points beyond the size of this cavity does not provide any benefit. We estimated that a cylindrical hole with a diameter of $15\,\text {mm}$ would be sufficient to accommodate the electrode cables.

As outlined in “Fenestration size” section, we assumed $30^{\circ }$ to be an ideal SCC entry angle. To determine a suitable configuration of mutual trajectories, we performed an optimization under the following constraints: (A) all trajectories must have a safety margin greater than $1\,\text {mm}$; (B) the SCC entry angles $\alpha _i$ must be less than $80^{\circ }$ to avoid retrograde insertion; and (C) the pairwise distance between any two drill entry points must be less than $12.5\,\text {mm}$.

Within these constraints, we minimize the objective function $\sum _i (\alpha _i - 30^{\circ })^2$, which penalized deviations from the ideal SCC entry angle. This formulation is effective in reducing large, unfavorable SCC entry angles.

### Feasibility analysis

We defined an *accessible* trajectory as one that satisfied a safety margin greater than $1\,\text {mm}$ and a SCC entry angle below $80^{\circ }$. Access to a target was *feasible*, if at least one accessible trajectory within the analyzed mastoid surface region was present. A case was feasible, if acessible trajectories for superior, lateral and posterior targets were present, that originated from within a circle of $15\,\text {mm}$ in diameter on the mastoid surface, according to “Mutual trajectory optimization” section. The *accessibility* of a case was the percentage of acessible trajectories.

## Results

Across all 30 evaluated cases, safe drill trajectories to the superior, lateral, and posterior semicircular canals were identified. Access with sufficient safety margins and favorable SCC entry angles was achievable from the majority of evaluated drill entry points.

We also found that in all cases, it was feasible to plan three safe trajectories, one to each semicircular canal, that originate from a single compact region on the mastoid surface. An overview of safety distances to all investigated structures for these mutually optimized trajectories is provided in Table 1. The ratio of accessible entry points and statistics on trajectory orientation can be found in Table 2. All results are further detailed below.

**Table 1:** Summary statistics (median, interquartile range and range, all values in millimetres) of the safety margins of the mutually optimized drill trajectories for all cases

Target Site	Superior Canal			Lateral Canal			Posterior Canal		
	Median	IQR	Range	Median	IQR	Range	Median	IQR	Range
Obstacle									
Superior canal	–			3.6	3.5-3.8	3.0-4.2	8.0	7.7-8.1	7.4-8.7
Lateral canal	3.0	2.9-3.1	2.6-3.5	–			2.9	2.5-3.1	2.3-3.2
Posterior canal	7.3	7.0-7.6	6.1-8.1	6.6	6.2-6.9	5.5-7.5	–		
Facial nerve	4.6	4.5-5.2	3.8-8.7	2.1	1.9-2.4	1.5-4.2	2.9	2.4-3.5	1.2-4.3
Chorda tympani	7.3	6.9-7.6	6.2-8.2	4.8	4.7-5.0	4.1-5.4	6.7	6.1-7.1	5.1-7.6
Lateral skull base	2.0	1.0-2.8	1.0-4.5	5.5	4.7-6.2	1.9-7.1	5.0	3.7-6.8	1.7-11
Sigmoid sinus	11	9.3-14	1.1-19	8.1	6.7-11	3.7-19	6.8	3.2-8.0	1.7-13
External auditory canal	9.2	8.3-12	7.7-18	6.3	6.0-8.6	5.1-18	8.1	7.2-10	5.7-16

### Safe trajectories

**Fig. 3:**
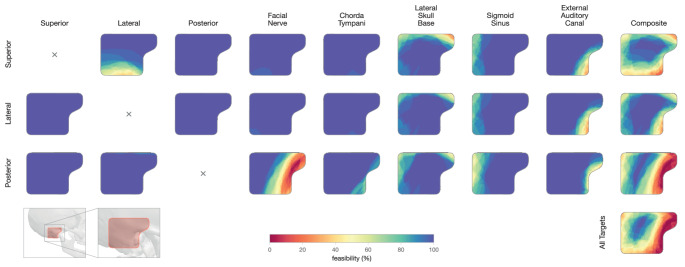
Accessibility matrix showing the feasibility of drill trajectories to each semicircular canal (rows) with respect to surrounding anatomical obstacles (columns), across all 30 cases. Color indicates the percentage of cases in which a given entry point maintains a safety margin of at least $1\,\text {mm}$. The rightmost column shows regions safe from all structures for each target, and the bottom-right map highlights entry regions that are simultaneously safe for access to all three canals

We identified safe trajectories within the evaluated entry region across all analyzed cases. Figure 3 shows the accessibility for all cases separately for all analyzed structures.

Most prominently, access was constrained anteriorly by the external auditory canal, superiorly by the lateral skull base, and posteriorly by the sigmoid sinus. For the superior canal, the lateral canal constrained inferior approaches, while access to the posterior canal was restricted anteriorly by the facial nerve. With a safety margin of $1\,\text {mm}$, the percentage of accessible drill entry points was 75% (IQR 15%) for the superior, 87% (IQR 10%) for the lateral and 59% (IQR 14%) for the posterior canal targets.

**Table 2:** Summary statistics (median, interquartile range, and range) for accessibility, angular and distance metrics

Target Site	Superior Canal			Lateral Canal			Posterior Canal		
	Median	IQR	Range	Median	IQR	Range	Median	IQR	Range
Accessibility									
2 mm safety margins to critical structures (% of entry points)	48	39–58	15–77	62	0–79	0–92	30	22–43	0–62
1 mm safety margins to critical structures (% of entry points)	77	64–87	30–98	91	83–95	64–99	61	51–67	23–87
0.5 mm safety margins to critical structures (% of entry points)	95	88–99	55–100	97	94–100	81–100	80	75–89	59–95
Drill Path Orientation (with 1 mm safety margin)									
Lowest accessible SCC entry angle (${}^{\circ }$)	61	56–66	53–79	3	0–7	0–19	11	4–14	0–25
Lowest accessible angular offset to CI drill direction (${}^{\circ }$)	0	0–3	0–17	0	0–1	0–9	0	0–1	0–20
Lowest accessible Mastoid entry point distance to CI entry point (mm)	7	4–9	1–16	7	4–8	1–16	7	5–9	1–18
SCC entry angle with CI drill direction (${}^{\circ }$)	77	70–84	60–102	39	33–51	25–94	37	30–49	20–83
Mutually Optimized Trajectory									
SCC entry angle (${}^{\circ }$)	61	56–67	53–79	30	30–30	30–33	30	30–31	30–41
Angular offset to CI drill direction (${}^{\circ }$)	30	17–38	3–79	28	19–40	3–80	26	15–35	7–62
Mastoid entry point distance to CI entry point (mm)	21	14–25	10–37	19	14–24	7–35	18	14–23	9–31
Drill canal length (mm)	31	27–35	20–39	28	26–31	18–38	31	28–35	21–38

At a safety margin threshold of $0.5\,\text {mm}$ (commonly applied in cochlear implantation), most drill entry points within the analyzed mastoid surface region supported safe access to the semicircular canals. Trajectories aligned with or close to the cochlear implant (CI) drill direction were generally feasible, though this resulted in high semicircular canal (SCC) entry angles for the superior canal. The proposed optimization strategy effectively selected the lowest accessible approach angle for the superior canal and identified trajectories to the lateral and posterior canals that remain close to the ideal approach angle of $30^{\circ }$

### Semicircular canal entry angles

**Fig. 4:**
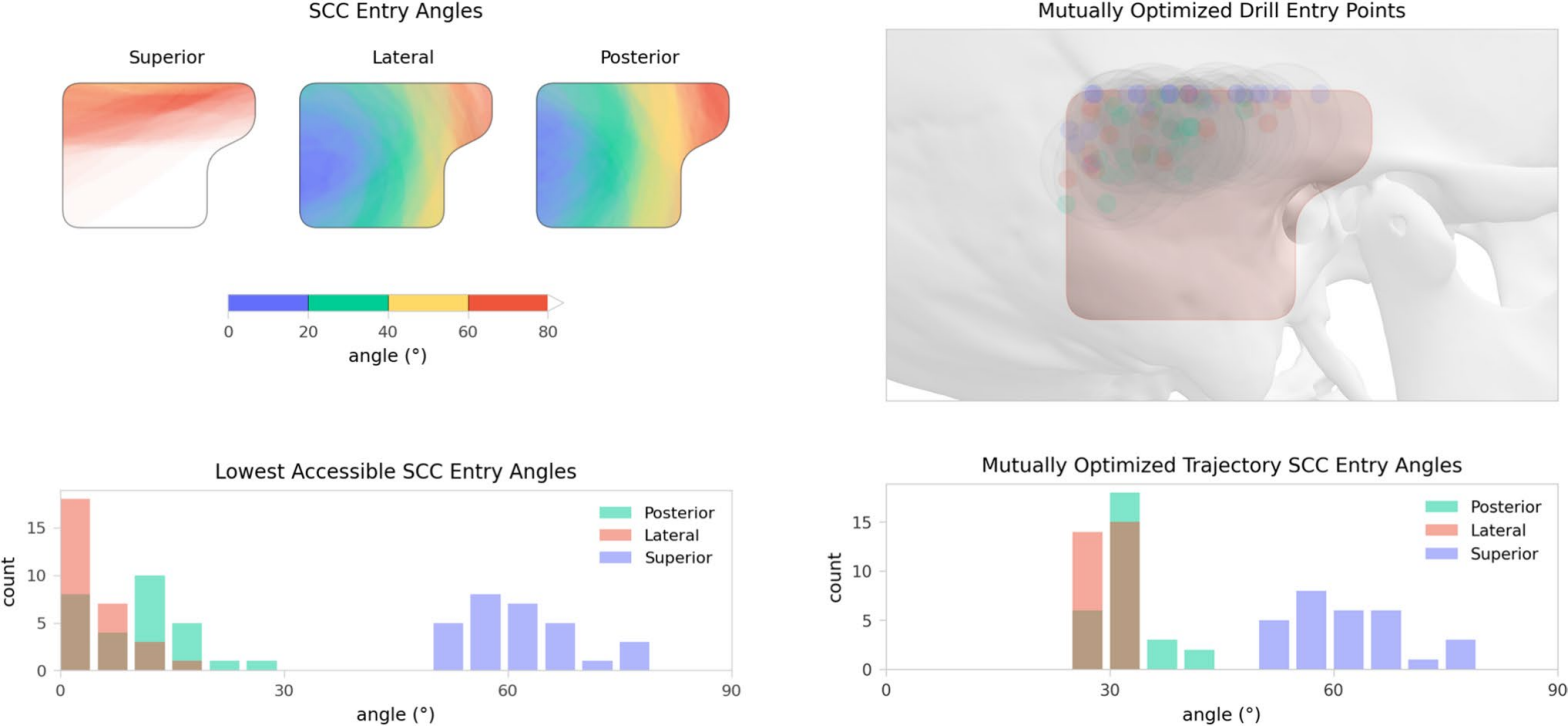
Top left: Distribution of the semicircular canal (SCC) entry angles for all analyzed cases. Tangential trajectories to the lateral and posterior targets typically led through the posterior section of the entry point area. Tangential trajectories to the superior canal led through the lateral skull base, with the lower half of the entry point area typically leading to right-angled or retrograde SCC entry angles. The observed lowest SCC entry angles (median $61^{\circ }$) were towards the superior border of the entry point area. Bottom left: Distribution of minimally accessible SCC entry angles. Top right: Optimized drill entry points for all cases, shown relative to the temporal bone of an exemplary patient. Bottom right: Corresponding SCC entry angles. For the superior canal, the algorithm typically selects the trajectory with the lowest accessible angle, while the selected lateral and posterior trajectories are close to the ideal $30^{\circ }$ approach angle

Figure 4 illustrates the distribution of SCC entry angles across all cases. Access to the lateral and posterior semicircular canals was consistently achievable within the entry area at shallow angles. In contrast, access to the superior semicircular canal required a larger SCC entry angle. Across the cohort, the median minimal accessible SCC entry angles were $61^{\circ }$ (IQR $10^{\circ }$) for the superior, $2^{\circ }$ (IQR $6^{\circ }$) for the lateral and $11^{\circ }$ (IQR $9^{\circ }$) for the posterior canal.

#### Varying the fenestration site

**Fig. 5:**
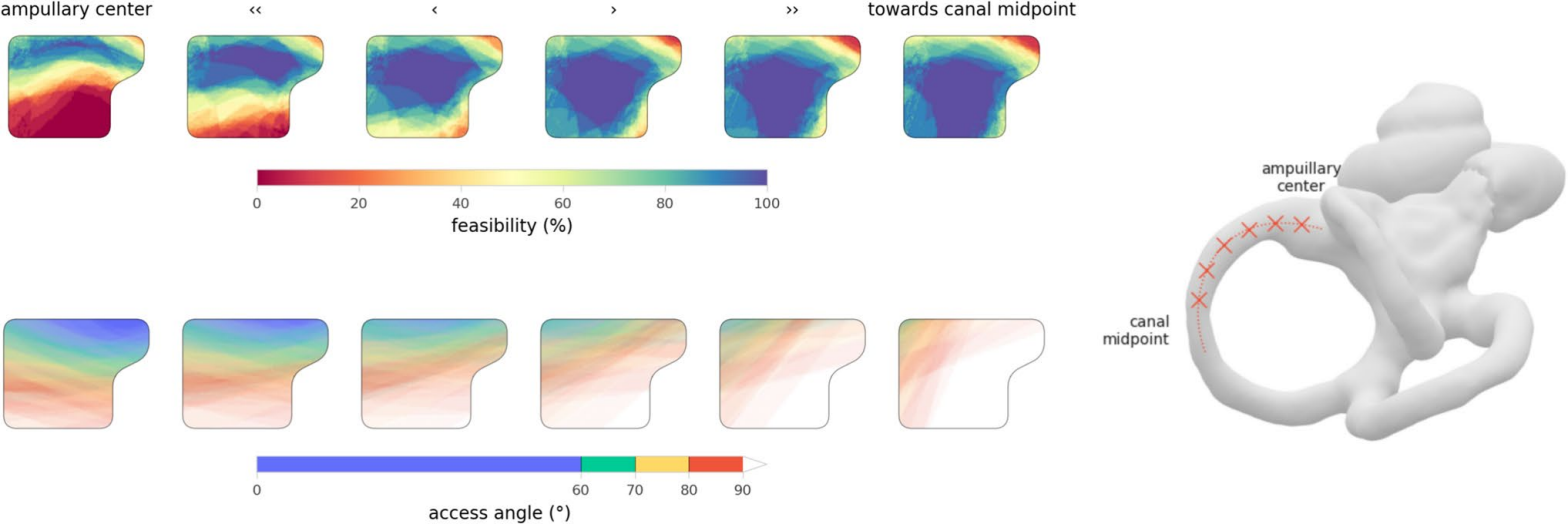
Sweep through six fenestration sites along the superior semicircular canal. A target positioned near the ampullary centre reduces the accessibility but improves the minimal SCC entry angle. As the fenestration site moves away from the ampulla, the minimal SCC entry angle steadily deteriorates, while accessibility improves only marginally beyond a distance of $2\,\text {mm}$ from the ampulla

Varying the fenestration site along the superior semicircular canal affected both accessibility and the SCC entry angle. Figure 5 illustrates the impact of shifting the fenestration site from direct ampullary access up to a distance of $6\,\text {mm}$ along the canal, evaluated across all cases. We observed that the lateral semicircular canal primarily constrained access when targeting the ampulla directly. The assessibility of all fenestration sites located more than $2\,\text {mm}$ from the ampulla effectively the same. However, the SCC entry angle progressively worsened with increasing distance from the ampulla.

#### Mutual trajectory optimization

Mutually optimal drill entry points obtained according to “Mutual trajectory optimization” section were feasible in all ears and are shown in Fig. 4. For all cases, the largest SCC entry angle occured into the superior canal, with a median value of $61^{\circ }$ (IQR $10^{\circ }$), while access to the other canals was essentially at the ideal angle with $30^{\circ }$ (IQR $0^{\circ }$) for the lateral and $30^{\circ }$ (IQR $1^{\circ }$) for the posterior canal.

## Discussion

In this study, we evaluated the anatomical feasibility of robotic keyhole access for intralabyrinthine vestibular implantation, using imaging data from 30 cases. Safe drill trajectories were identified for all semicircular canals across the cohort. Shallow SCC entry angles were consistently achievable for the lateral and posterior canals. Access to the superior canal required steeper angles but remained within a feasible range for all cases. We found that increasing the distance of the fenestration site from the ampullary centre along the superior canal had minimal impact on accessibility, but led to progressively steeper SCC entry angles. Furthermore, we identified mutually optimized trajectories that originate from a compact region on the mastoid surface in all cases, supporting the practical viability of a minimally invasive keyhole approach.

### Anatomical constraints

Our findings demonstrate that intralabyrinthine keyhole access is feasible for all semicircular canals, with safety margins that are consistently within clinically acceptable limits. Anteriorly, trajectories to the superior and lateral semicircular canals are constrained by the external auditory canal, and for the posterior canal by the facial nerve. As all trajectories pass posterior to the facial nerve, the chorda tympani does not impose additional constraints. Superiorly, trajectories are limited by the lateral skull base, and posteriorly by the sigmoid sinus. The canals themselves do not contribute to further constraints except for the lateral canal which limits inferior access to the superior canal in some anatomies. Overall, the majority of evaluated trajectories within the $30\,\text {mm} \times 30\,\text {mm}$ drill entry point area proved to be accessible in all cases.

Image guided robotic access to the inner ear is already in clinical use for cochlear implantation [2, 3], where the drill trajectory is required to pass between the facial nerve and the chorda tympani. We observed that the spatial constraints for vestibular access are in comparison less restrictive. In most anatomies, safety margins of $1\,\text {mm}$ or greater were readily achievable. Given the documented accuracy of image-guided robotic drilling of $0.15\,\text {mm}$ [17], these results even suggest that current rigorous intraoperative safety protocols could be reconsidered. In particular, the need for an intraoperative computed tomography scan midway through the procedure to verify trajectory accuracy might be omitted for cases with adequate safety margins.

### Semicircular canal entry angles

Unlike cochlear implantation, where the round window provides a well-defined surgical target, vestibular implantation allows for more flexibility in choosing the fenestration site along the canal. We hypothesized that direct drilling into the ampulla should be avoided to prevent damage to the sensitive sensory epithelia. Instead, we proposed accessing the canal at a short distance from the ampulla, and subsequently advancing the electrode along the canal toward the ampullary nerve.

This approach imposes two competing requirements: on one hand, a shallow SCC entry angle is needed to facilitate smooth advancement of the electrode along the canal; on the other hand, a fully tangential approach results in an excessively large exposure of the canal lumen (see Fig. 2 for an illustration of this effect). Based on our analysis (see “Fenestration size” section), we proposed an ideal SCC entry angle of $30^{\circ }$, which offers a suitable compromise between these factors.

Safe trajectories with SCC entry angles close to this ideal are consistently achievable for the lateral and posterior semicircular canals. In contrast, the superior canal is oriented in the sagittal plane, precluding low-angle access from the mastoid surface in typical anatomies, as corresponding trajectories would lead through the lateral skull base (the distribution of SCC entry angles are visualised in Fig. 4, and a typical example shown in Fig. 1, (E, F)). In our cohort, we found that the superior canal can be approached at a median angle of $61^{\circ }$. Even in the worst observed case, with an accessible SCC entry angle of $79^{\circ }$, electrode insertion is likely feasible without risking a retrograde trajectory.

In this context, it should be considered whether the SCC entry angle can be improved by changing the position of the fenestration along the canal. Our analysis shows that SCC entry angles become progressively worse as the fenestration site is moved away from the ampulla (see Fig. 5). In contrast, the accessibility improves only marginally beyond a distance of $2\,\text {mm}$ from the ampullary centre. Based on this trade-off, we conclude that selecting a fenestration site approximately $2\,\text {mm}$ from the electrode target offers a reasonable compromise between optimizing the SCC entry angle and maintaining sufficient accessibility. Ultimately, individual positional adjustments may be appropriate for clinical application.

Van de Berg et al. have suggested that the electrode should extend into the canal approximately one third of its length to ensure optimal fixation and positioning [21]. As the robotic keyhole access further confines the electrode within a narrow trajectory, the effective intralabyrinthine length may be slightly reduced. Based on this, our chosen default distance of $2\,\text {mm}$ represents a reasonable compromise. Ultimately, the balance between achieving favourable SCC entry angles and maximising implant fixation can be tailored to suit each individual anatomy.

### Mutual trajectory optimization

Selecting the optimal drill trajectory for each semicircular canal independently typically resulted in a wide spatial spread of drill entry points, which is not suitable for implant placement. Even under a keyhole access paradigm, a simple cortical mastoidectomy is needed to accommodate the excess electrode lead cables. All three drill trajectories should therefore enter within this confined area. In the present study, we assumed a circular mastoidectomy with a diameter of $15\,\text {mm}$, and jointly optimized the three trajectories to minimize SCC entry angle deviations under this constraint.

Specifically, we minimized the sum of the squared differences between each trajectory’s SCC entry angle and the ideal angle. The quadratic formulation of this objective penalizes large deviations more heavily, particularly affecting the trajectory to the superior canal, where the largest SCC entry angles are typically observed. As a result, the optimization tends to select the lowest feasible SCC entry angle for the superior canal, while identifying nearby trajectories for the lateral and posterior canals that both satisfy the mutual distance constraint and remain close to the ideal angle.

We found that this mutually optimized drill trajectory selection was feasible in all evaluated cases. The resulting mastoidectomy region was typically located in the superior or posterosuperior portion of the analyzed drill entry point area, as can be observed in Fig. 4.

### Implications for future research and clinical applications

Currently, implant electrode placement of vestibular implants through a canal fenestration typically relies on intraoperative imaging, as the surgeon cannot visually estimate the exact distance between the fenestration and the electrode target site. One advantage of a robotically drilled access is the precisely known geometry of the drill trajectory and its geometric relation to the electrode target site. This opens up the potential for robot-assisted electrode placement, allowing implants to be positioned accurately at the target location, without additional imaging. In cochlear implantation, robot-assisted insertion is clinically available and has been shown to potentially reduce insertion trauma, with lower pressure transients and lower forces applied to intracochlear structures [1]. Similar benefits may be achievable for vestibular implantation with a potentially lower risk of postoperative hearing loss and tinnitus. Such keyhole procedure might also reduce the risk of postoperative electrode displacement since a large portion of the electrode lead remains in the drilled canal rather than being free-floating in the mastoid cavity. By improving the accuracy of electrode placement through a known distance length to the ampulla, we expect a lower risk of inadequate electrode placement in the future.

Beyond electrode insertion, the demonstrated feasibility of robotic keyhole access to the vestibular system opens possibilities for broader clinical applications. Minimally invasive access may support future procedures such as the treatment of superior canal dehiscence syndrome (SCDS), canal plugging for benign paroxysmal positional vertigo (BPPV), and selective vestibular neurectomy. In future work, we plan to systematically assess the feasibility of these interventions using the robotic keyhole approach. Translating these anatomical results into routine surgery will also require addressing practical implementation challenges, including adaptation of instruments for keyhole vestibular access, optimisation of patient positioning and fiducial placement, and integration of multiple drill trajectories into a streamlined planning and execution workflow.

### Limitations

In this work, we focused on assessing the anatomical feasibility of robotic vestibular access. The analysis did not incorporate the kinematic constraints of the robotic system, which may affect the feasibility of certain trajectories. To mitigate this, we defined a reasonably compact drill entry point area, which limited angular deviations from the typical cochlear implant drill direction. In our data, the angular deviation between vestibular drill trajectories and the planned cochlear drill trajectory was typically below $30^{\circ }$, which is likely manageable through minor adjustments in patient positioning. We therefore expect that robotic access is feasible through the analyzed drill entry points.

The currently available robotic system is optimized for cochlear implantation, and to permit clinical use for intralabyrinthine access, adaptations to the user interface and surgical workflow are required. This includes extending current planning software, which is designed for a single cochlear trajectory, to handle multiple vestibular trajectories within the same operative setup, ensuring that registration is maintained across all planned drill paths. In particular, safety margins towards the semicircular canals, sigmoid sinus and lateral skull base must be computed by the planning software, as these structures are not typically modelled for cochlear implantation but are critical for vestibular access.

Future work should evaluate feasible placement of the patient tracking marker. For cochlear implantation, this marker is typically positioned posterior to the drill entry point, which might interfere with typical vestibular drill trajectories. Ultimately, the capability of the robotic system to reach the planned trajectories must be validated in a realistic experimental or clinical setting. As a preliminary step, we have already performed keyhole access to the semicircular canals in a pilot study using cadaver specimens.

Next, whether threading the electrode cables through a keyhole access into the semicircular canals is feasible remains to be tested and validated in future studies. Generally, the procedure is very similar to cochlear implantation, with comparable approach angles and electrode design. However, in cochlear implantation, the round window can be visually accessed through the middle ear, while direct visual inspection of the fenestration site is not possible for vestibular access. In addition, the guide tube design that prevents the cochlear implant electrode array from catching on mastoid air cells may require further adaptation to ensure smooth and accurate insertion into the semicircular canals. One potential strategy is a hybrid approach that begins with a conventional mastoidectomy to allow standard surgical exposure. Robotic drilling would then be used to create precise fenestrations in the skeletonized semicircular canals. This approach would eliminate the need for low SCC entry angles, thereby simplifying both access and electrode insertion.

Finally, in this study, we assumed a generic vestibular implant designed to stimulate three sites within the vestibular system. Some implant designs additionally include a cochlear implant electrode array and/or components for otolith stimulation. In the case of cochlear integration, the general requirements and constraints of robotic cochlear implantation apply. In defining mutually optimized drill trajectories for vestibular access, we did not account for the spatial relationship to the cochlear drill entry point. In practice, the surgeon would need to drill a connecting channel on the mastoid surface from the cochlear drill entry point to the cortical mastoidectomy, which mirrors the standard procedure used in current robotic cochlear implantation, where the channel is drilled up to the implant bed [22]. In our data, the required distance from the cochlear drill entry point to the mastoidectomy is typically below $20\,\text {mm}$ (see Table 2). Therefore, we do not anticipate any major limitations for implant integration in this context.

## Conclusion

This study demonstrates the anatomical feasibility of robotic keyhole access to all three semicircular canals for intralabyrinthine vestibular implantation. Using high-resolution imaging data from 30 cases, we identified safe trajectories with sufficient safety margins. Low SCC entry angles can be achieved, particularly for the lateral and posterior canals. The superior canal presents the greatest geometric constraints, although access remains feasible in all cases.

Importantly, we have demonstrated that all three drill trajectories can be targeted through a compact retroauricular area on the mastoid surface, allowing the use of a single, simple cortical mastoidectomy to accommodate the implant’s electrode lead cables.

These results support the wider use of image-guided robotic systems for vestibular implantation. With further development, particularly in refining electrode delivery tools and adapting robotic workflows, this technique may offer a minimally invasive, precise and reliable solution for treating and restoring vestibular function in affected patients.
